# Percutaneous drainage under the control of ultrasound of the left-sided subphrenic abscess after gastrectomy: A case report

**DOI:** 10.1016/j.amsu.2019.09.009

**Published:** 2019-09-20

**Authors:** Radmila Karpova, Evgeniya Kirakosyan, Tatyana Khorobrykh, Alexander Chernousov

**Affiliations:** a Department of Faculty Surgery №1, Federal State Autonomous Educational Institution of Higher Education I.M. Sechenov First Moscow State Medical University of the Ministry of Health of the Russian Federation (Sechenov University), Russia; b International School “Medicine of the Future”, Federal State Autonomous Educational Institution of Higher Education I.M. Sechenov First Moscow State Medical University of the Ministry of Health of the Russian Federation (Sechenov University), Russia; c Academic of Russian Academy of Science, head of the Department of Faculty Surgery №1, Federal State Autonomous Educational Institution of Higher Education I.M. Sechenov First Moscow State Medical University of the Ministry of Health of the Russian Federation (Sechenov University), Russia

**Keywords:** Subphrenic abscess, Fistula, Percutaneous drainage, Fibrin glue, Control of ultrasound and X-ray

## Abstract

**Introduction:**

Abdominal abscesses are one of the frequent and dangerous postoperative complication. They occur as a result of failure of seams esophagojejunal anastomosis after gastrectomy (17%), perforation of gastric and duodenal ulcers (26.8%), splenectomy (25.4%), failure of biliodigestive anastomoses (23.8%), inadequate drainage of the subphrenic space (22.2%), acute pancreatitis (14%). Left-sided subphrenic abscesses are the most common of them.

**Case presentation:**

We present a patient with the left-sided subphrenic abscess, formed as a result of insolvency of the esophagojejunal anastomosis after gastrectomy and splenectomy, which underwent percutaneous drainage under the control of ultrasound and X-ray. Sanitation of the abscess cavity and the introduction of fibrin glue into it made it possible to close the fistula and heal the patient.

**Discussion:**

The described case shows that the rehabilitation of the abscess and the injection of fibrin glue into it, made it possible to avoid surgery, eliminate the abscess and close the connection with the esophagojejunal anastomosis in a short time.

**Conclusion:**

Percutaneous drainage under the control of ultrasound made it possible to avoid surgery and heal the patient with the left-sided subphrenic abscess in a short time. Fistula treatment with fibrin glue is not only effective, but is also less risky than surgery.

## Highlights

• Abdominal abscesses are frequent and dangerous postoperative complications, and left-sided subphrenic abscesses are the most common of them.
• Percutaneous drainage made it possible to avoid surgery and heal the left-sided subphrenic abscess in a short time.
• Fistula treatment with fibrin glue is not only effective, but is also less risky than surgery.

## 1 Introduction

Abdominal abscesses are one of the frequent and dangerous postoperative complications. According to literature, they occur in 0.6% of planned and 1.5% of emergency surgical interventions. The most common cases are left-sided subphrenic abscesses (LSA), which are formed as a result of failure of seams esophagojejunal anastomosis after gastrectomy (17%), perforation of gastric and duodenal ulcers (26.8%), splenectomy (25.4%), failure of biliodigestive anastomoses (23.8%), inadequate drainage of the subphrenic space (22.2%), acute pancreatitis (14%). Today, an effective treatment for LSA is its puncture and drainage under the control of ultrasound and X-ray. The effectiveness of this method ranges from 60 to 92% [[1], [2], [3]]. However, this method does not always allow to cure a patient with a large abscess that has a connection with hollow organs or ductal structures: in 33% of cases this fistula does not heal on its own and requires surgical intervention [4,5]. The mortality rate is quite high - from 27 to 98% [6].

We present a patient with LSA, formed as a result of insolvency of the esophagojejunal anastomosis after gastrectomy and splenectomy, which underwent percutaneous drainage under the control of ultrasound and X-ray. Sanitation of the abscess cavity and the introduction of fibrin glue into it made it possible to close the fistula and heal the patient.

## 2 Case presentation

Patient S., 62 years old, was at the Sechenov University Clinical Hospital No. 1 with a diagnosis of left-sided subphrenic abscess, condition after gastrectomy and splenectomy for gastric cancer (T3N1M0).

**Fig. 1:**
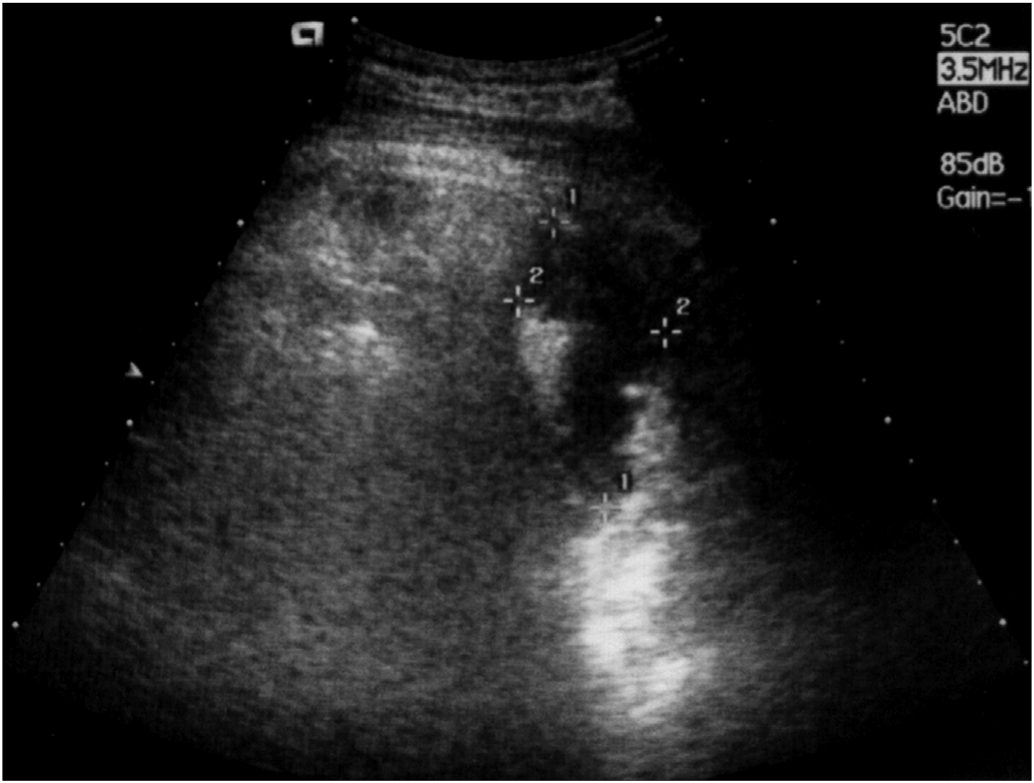
Ultrasound scan of the left-sided subphrenic abscess (indicated by crosses).

Gastrectomy, splenectomy, abdominal cavity drainage had been performed. On the 3rd day after the surgery, the ultrasound examination of the abdominal cavity revealed a fluid accumulation of 35 ml in left subphrenic space. No signs of abscess were detected. Drainages were removed from the abdominal cavity. However, on the 14th day the body temperature increased up to 39 °C, chills, epigastric pain, leukocytosis up to (17*10^9^/l) were recorded. Abdominal ultrasound revealed LSA volume of about 75 ml (Fig. 1) [insert Fig. 1.]. An X-ray examination (contrast radiography) indicated consistent of esophagojejunal anastomosis.

**Fig. 2:**
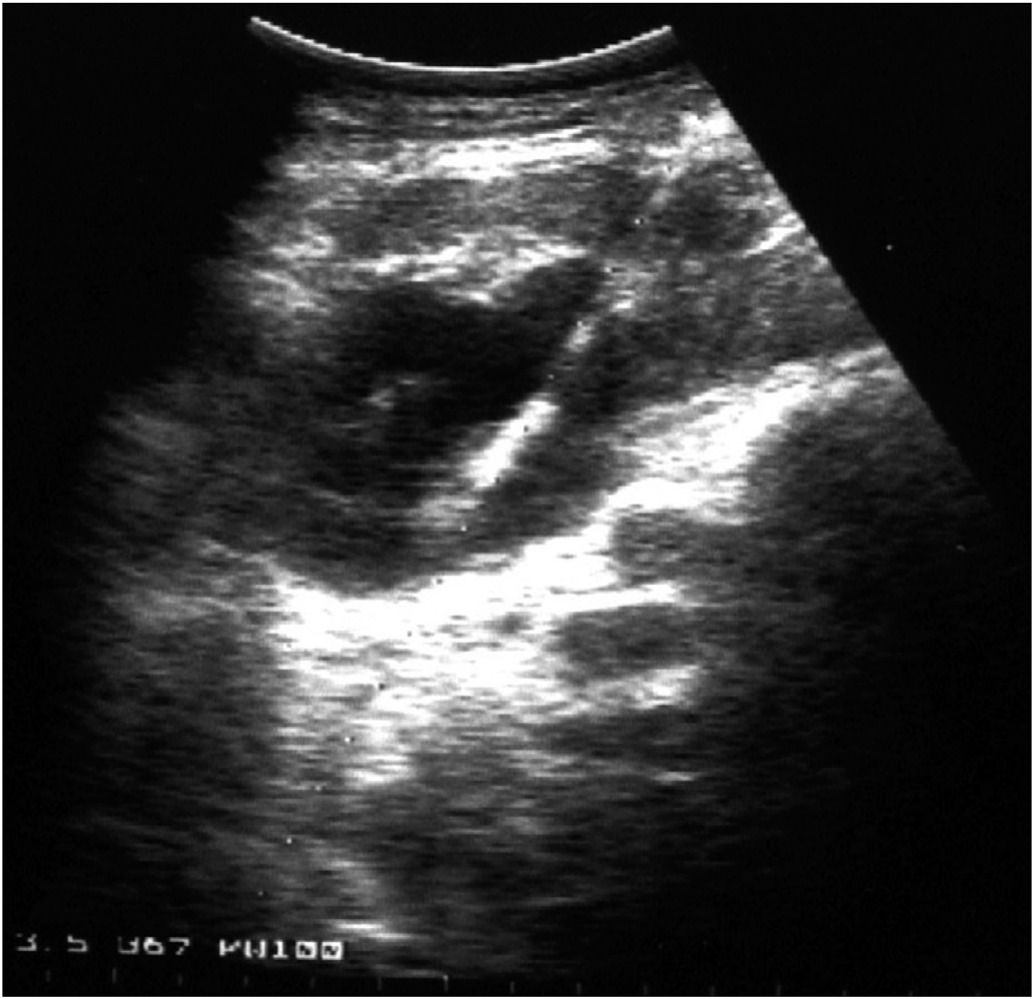
Ultrasound scan of percutaneous drainage of left-sided subphrenic abscess by the stylet-catheter.

**Fig. 3:**
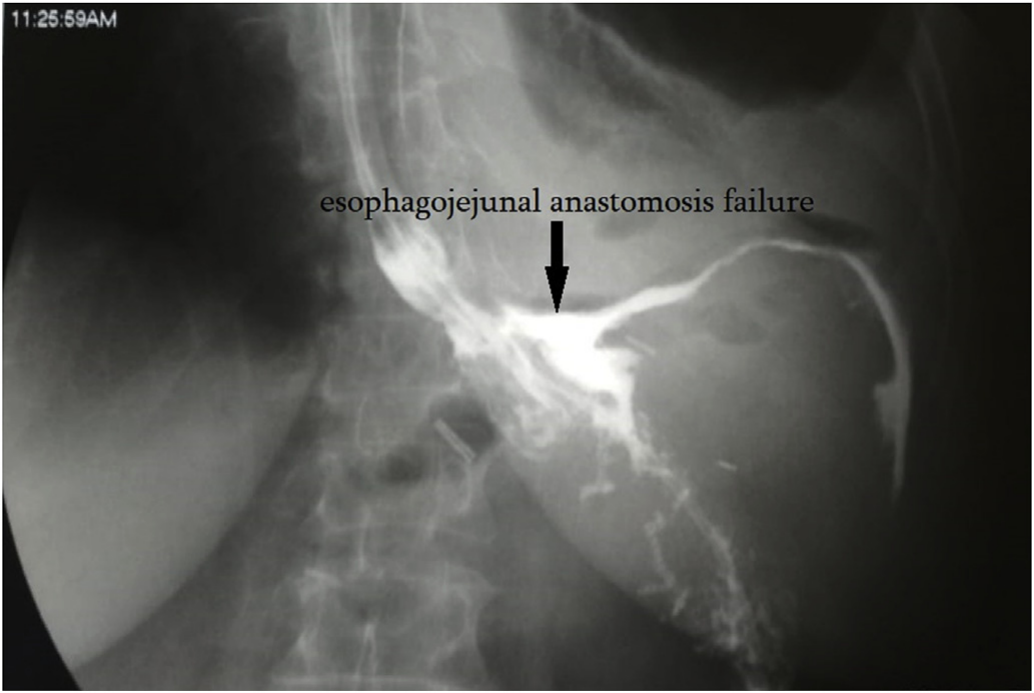
Contrast X-ray: failure of esophagojejunal anastomosis with the formation of fistula.

Percutaneous drainage of LSA was performed under the control of ultrasound and X-ray by using the stylet-catheter method (universal curved drainage, material Ultrathane, the distal tip of Intro-Tip, hydrophilic coating AQ) (Fig. 2) [insert Fig. 2.]. 55 ml of pus were evacuated and sent for bacteriological and cytological examination. While inserting the contrast, a fistula was found leading to the esophagojejunal anastomosis (Fig. 3) [insert Fig. 3.]. The cavity was washed with antiseptic solutions. Bacteriological examination revealed *Staphylococcus aureus* 10^7^, sensitive to metronidazole. Subsequently, the abscess cavity was washed with metronidazole. Despite the sanitation of the abscess, fistula was visible during contract insertion. To close the fistula, fibrin glue was injected into the abscess cavity, 4 ml three times every second day. The control insertion of contrast showed no fistula. The residual abscess cavity was 5 ml. The drainage was removed.

As a result of microfistula in the area of esophagojejunal anastomosis, the contents of the gastrointestinal tract entered the abscess cavity and supported the inflammatory process in it. The sanitation of the abscess cavity and insertion of fibrin glue made it possible to close the fistula and heal the abscess.

## 3 Discussion

Percutaneous drainage is definitely the most effective, less traumatic method for the treatment of subphrenic abscesses. However, there are difficulties in choosing a puncture pathway that runs close to or through the pleural sinus, as well as through the loop of the intestine. In case of a bad choice of a puncture pathway, postoperative complications may develop, e.g. pneumothorax (50%), empyema (10%), bleeding (0.6%). Mortality varies from 10 to 90%. In such cases, surgery may be required [1,7,8].

According to the literature, 15–51% of patients with abdominal abscess are diagnosed with a connection between the abscess cavity and the damaged intestine. This greatly complicates the tactics of treatment and extends the bed-day. Lack of esophagojejunal anastomosis after gastrectomy and the formation of fluid buildup under the diaphragm occurs in 4–15% of cases. Percutaneous drainage and sanitation of such abscesses is not effective in 12–33% of cases [4,5].

Nowadays, fibrin glue is widely used in surgical practice. It is used in various fields of medicine: for the treatment of tracheobronchial and tracheoesophageal, gastrointestinal, rectovaginal and perineal fistulas, etc. [9]. Fibrin glue has been proven to contain pro- and anti-inflammatory cytokines, growth factors that contribute to tissue regeneration in the area of its injection [4,[10], [11], [12]]. It has been proven both in the experiment and in the clinic that fibrin glue supports tissue regeneration saving the physiological ratio of the parenchyma and stroma, without proliferation of connective tissue [13].

The described case shows that the rehabilitation of the abscess and the injection of fibrin glue into it, made it possible to avoid surgery, eliminate the abscess and close the connection with the esophagojejunal anastomosis within 14 days.

It should be noted that the average bed-day with percutaneous drainage of subphrenic abscesses without fistula is 13.2 days [1,14].

## 4 Conclusion

Percutaneous drainage under the control of ultrasound made it possible to avoid surgery and heal the patient with the left-sided subphrenic abscess in a short time. Fistula treatment with fibrin glue is not only effective, but is also less risky than surgery. This is a safe method that does not require endotracheal anesthesia, a skin incision, and a long bed-day [9].

## Informed consent of patient

Written informed consent was obtained from the patient for his anonymized information to be published in this article. The patient provided his informed consent for the publication of his clinical details and any accompanying images about this case report.

## Provenance and peer review

Not commissioned, externally peer reviewed.

## Ethical approval

NA.

## Sources of funding

This research received no specific grant from any funding agency in the public, commercial, or not-for-profit sectors.

## Author contribution

Radmila Karpova, Evgeniya Kirakosyan, Tatyana Khorobrykh, Alexander Chernousov performed the procedure, wrote the manuscript and are responsible for the information.

## Conflicts of interest

The Authors declare that there is no conflict of interest.

## Registration of research studies

1. Name of the registry: NA
2. Unique Identifying number or registration ID: NA
3. Hyperlink to the registration (must be publicly accessible):

## Guarantor

Radmila Karpova, Evgeniya Kirakosyan, Tatyana Khorobrykh, Alexander Chernousov.
